# Impact of treatment interval between neoadjuvant immunochemotherapy and surgery in lung squamous cell carcinoma

**DOI:** 10.1186/s12885-024-12333-3

**Published:** 2024-05-13

**Authors:** Chen Gu, Xiao Teng, Xuqi sun, Jiacong Liu, Ziyue Zhu, Lichen Zhang, Zhigang Wu, Rui Zou, Jinghua Pang, Xiayi Lyu

**Affiliations:** 1 Thoracic Surgery, The First Affiliated Hospital, Zhejiang University School of Medicine, 79 Qingchun Road, Shangcheng District, Hangzhou, 310000 China; 2 Zhejiang University School of Medicine, Huangzhou, China; 3 Thoracic Surgery, Fenghua People’s Hospital, Ningbo, China; 4 Key Laboratory of Clinical Evaluation Technology for Medical Device of Zhejiang Province, Hangzhou, China

**Keywords:** Lung squamous cell carcinoma, Neoadjuvant immunochemotherapy, Treatment interval, Survival outcome

## Abstract

**Objective:**

The optimal timing for surgery following neoadjuvant immunochemotherapy for lung squamous cell carcinoma appears to be a topic of limited data. Many clinical studies lack stringent guidelines regarding this timing. The objective of this study is to explore the effect of the interval between neoadjuvant immunochemotherapy and surgery on survival outcomes in patients with lung squamous cell carcinoma.

**Methods:**

This study conducted a retrospective analysis of patients with lung squamous cell carcinoma who underwent neoadjuvant immunochemotherapy between January 2019 and October 2022 at The First Affiliated Hospital, Zhejiang University School of Medicine. Patients were divided into two groups based on the treatment interval: ≤33 days and > 33 days. The primary observational endpoints of the study were Disease-Free Survival (DFS) and Overall Survival (OS). Secondary observational endpoints included Objective response rate (ORR), Major Pathological Response (MPR), and Pathological Complete Remission (pCR).

**Results:**

Using the Kaplan-Meier methods, the ≤ 33d group demonstrated a superior DFS curve compared to the > 33d group (*p* = 0.0015). The median DFS for the two groups was 952 days and 590 days, respectively. There was no statistical difference in the OS curves between the groups (*p* = 0.66), and the median OS was not reached for either group. The treatment interval did not influence the pathologic response of the tumor or lymph nodes.

**Conclusions:**

The study observed that shorter treatment intervals were associated with improved DFS, without influencing OS, pathologic response, or surgical safety. Patients should avoid having a prolonged treatment interval between neoadjuvant immunochemotherapy and surgery.

**Supplementary Information:**

The online version contains supplementary material available at 10.1186/s12885-024-12333-3.

## Introduction

Approximately 23% of non-small cell lung cancers are characterized as lung squamous cell carcinoma(LUSC) [1]. Survival rates for LUSC remain suboptimal, leading to unsatisfactory clinical outcomes. LUSC is known to be highly immunogenic [2]. The use of preoperative programmed cell death protein 1 (PD-1) or its ligand PD-L1, either as monotherapy or in combination with chemotherapy, has been associated with improved outcomes in LUSC [3]. Nonetheless, numerous questions remain concerning the application and efficacy of immunochemotherapy.

Liu et al. [4]demonstrated that a short interval (4–5 days) between the initiation of neoadjuvant immunotherapy and resection of the primary tumor is crucial for achieving optimal therapeutic efficacy. Prolonging the duration (10 days) or shortening it (2 days) eliminated the effectiveness of immunotherapy. The finding suggests that the treatment interval can significantly influence therapeutic efficacy. The optimal timing for surgery following neoadjuvant immunochemotherapy often seems overlooked. Many clinical studies lack a strict definition regarding this interval, with durations reported ranging from 21 to 49 days post the last neoadjuvant treatment [5–10].

Consequently, this study aims to examine the influence of the treatment interval between neoadjuvant immunochemotherapy and surgery on the prognosis of patients diagnosed with LUSC.

## Methods

This study retrospectively analyzed patients with stage IB-IIIB (T3N2, T4N2) LUSC who underwent neoadjuvant immunochemotherapy at The First Affiliated Hospital, Zhejiang University School of Medicine between January 2019 and October 2022.All the patients received between 2 and 4 cycles of neoadjuvant immunotherapy in combination with platinum-based doublet chemotherapy (comprising a platinum agent and paclitaxel) before surgery. The most recent follow-up for this study took place in July 2023.

We collected patients’ basic information, tumor response to neoadjuvant treatment, adverse events related to neoadjuvant therapy, extent of tumor regression, survival status, and other data through the hospital’s electronic medical record system and telephone follow-up. Preoperative and postoperative staging was conducted in accordance with the 8th edition of the American Joint Committee on Cancer (AJCC) and Lung Cancer Staging Manual’s Tumor, Lymph Node, and Metastasis (TNM) staging system [11]. The Charlson Comorbidity Index (CCI) was used to quantify patients’ comorbidities [12]. Charlson also proposed a CCI scoring standard that includes age weight [13]. After adding the score for comorbidities, the age-adjusted CCI (aCCI) score is obtained. Based on the range of the aCCI score, the severity of comorbidities is divided into three levels: none/mild comorbidities (aCCI score of 0–1), moderate comorbidities (aCCI score of 2–3), and severe comorbidities (aCCI score ≥ 4). Adverse events related to neoadjuvant treatment were evaluated based on the National Cancer Institute Common Terminology Criteria for Adverse Events (NCI-CTCAE) version 5.0 [14]. We evaluated the extent of tumor response to neoadjuvant treatment using the Response Evaluation Criteria in Solid Tumors (RECIST 1.1) [15], which is a standard criterion for assessing the efficacy of solid tumors. Complete Remission (CR): The complete disappearance of all target lesions, with no residual evidence of disease. Partial Remission (PR): A reduction in the sum of the longest diameters of target lesions by at least 30%. Progression Disease (PD): An increase of at least 20% in the sum of the longest diameters of target lesions or the appearance of new lesions.Stable Disease (SD): A status where changes fall between partial remission and progression [16]. The Objective Response Rate (ORR) is calculated as the sum of individuals achieving complete remission and partial remission, divided by the total number of individuals. All patients underwent PET-CT examination before neoadjuvant treatment. All patients underwent EBUS or biopsy before neoadjuvant treatment.

The treatment interval is defined as the duration between the last date of drug treatment and the date of surgery. Based on this interval, patients were divided into two groups: the < = 33 days group and the > 33 days group. The primary endpoints of this study were Disease-Free Survival (DFS) and Overall Survival (OS). The secondary endpoints included Objective Response Rate, Major Pathological Response (MPR), and Pathological Complete Remission (pCR). DFS is defined as the duration between the date of surgery and the date of the event occurrence. OS is defined as the duration between the date of the first neoadjuvant treatment and the date of the event occurrence. MPR was defined as 10% or fewer viable tumor cells in the resected primary tumor, and the pCR was defined as the removal of carinal tissues and dissected lymph nodes without any viable tumor [16, 17].

Patients meeting the following criteria were included in this study: (1) Age between 18 and 80 years. (2) Diagnosed with potentially resectable lung cancer confirmed by imaging, pathological histology, or cytology. Patients requiring neoadjuvant treatment as per standard diagnostic and therapeutic protocols for lung cancer prior to curative surgery. (3) ECOG performance status score of 0–1. (4) No prior treatment for lung cancer, including surgery, chemotherapy, radiotherapy, targeted therapy, hormone therapy, or immunotherapy.

Patients with any of the following conditions were excluded: (1) Lack of essential pre-treatment imaging assessment. (2) Presence of distant organ metastasis.

We performed intergroup analysis using t-tests, Mann-Whitney U tests, chi-square tests, or Fisher’s exact test. Analysis was conducted using the Cox regression model and logistic regression. We compared DFS and OS between groups using Kaplan-Meier methods and the log-rank test. All statistical tests in this study were two-tailed, with significance considered at a P-value < 0.05. All statistical analyses were performed using R software (version 4.2.1).

The study was approved by institutional ethics board of The First Affiliated Hospital, Zhejiang University School of Medicine (No. 2023 − 0472) and individual consent for this retrospective analysis was waived.

## Results

This study encompassed a total of 204 participants, with a median treatment interval of 33 days. In the < = 33 days group, there were 108 people, and the median treatment interval was 29 days; in the > 33 days group, there were 96 people, and the median treatment interval was 38 days. The treatment intervals of the two groups showed a bimodal distribution and there was a statistical difference (*p* = 0). The cohort consisted of 199 males (97.5%) and 5 females (2.5%). Males had a median age of 65 years, whereas for females, the median age was 66 years.

**Fig. 1:**
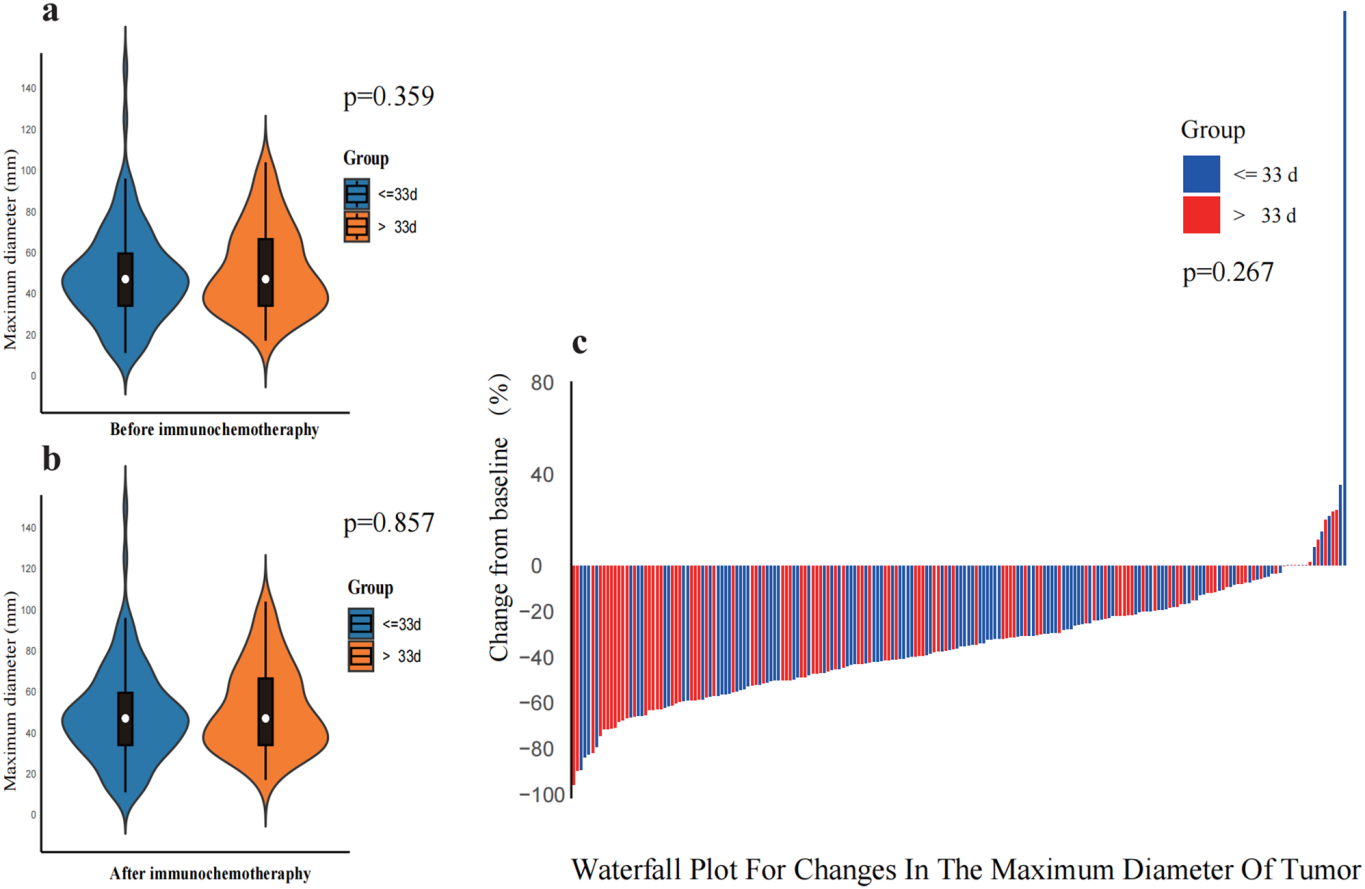
(**a**) Distribution of maximum tumor diameter in patients before treatment, *p* = 0.359. (**b**) Distribution of maximum tumor diameter in patients after neoadjuvant immunochemotherapy, *p* = 0.857. (**c**) Change in maximum tumor diameter after neoadjuvant immunochemotherapy in both groups, *p* = 0.267

There was no statistical difference in the aCCIs scores between the two groups. Moreover, the median initial tumor diameter was consistent at 47 mm for both groups, again showing no statistically significant variance (*p* = 0.359)(Fig. 1). Detailed baseline information can be found in Table 1.

**Table 1:** Comprehensive data about neoadjuvant immunochemotherapy

Variables	Group		*p*
	<=33d	> 33d	
**Clinical stage**			0.507
IB	13(12%)	17(17.7%)	
IIA	6(5.6%)	7(7.3%)	
IIB	15(13.90%)	18(18.8%)	
IIIA	40(37%)	29(30.2%)	
IIIB	34(31.5%)	25(26%)	
**Treatment cycle**			0.58
2	51(47.2%)	42(43.8%)	
3	26(24.1%)	20(20.8%)	
4	31(28.7%)	34(35.4%)	
**Immunotherapy Drugs**			0.139
Camrelizumab	20(18.7%)	18(18.8%)	
Pembrolizumab	20(18.7%)	20(20.8%)	
Durvalumab	0(0%)	1(1%)	
Nivolumab	19(17.8%)	7(7.3%)	
Tislelizumab	35(32.7%)	30(31.3%)	
Sintilimab	13(12.1%)	20(20.8%)	
yc-stage			0.576
IA	21(19.4%)	24(25%)	
IB	11(10.2%)	5(5.2%)	
IIA	23(21.3%)	15(15.6%)	
IIB	0(0%)	0(0%)	
IIIA	45(41.7%)	39(40.6%)	
IIIB	7(6.5%)	12(12.5%)	
IIIC	1(0.9%)	1(1%)	
Maximum diameter of tumor after treatment, P50(P25,P75),mm	27.23(19.12,38.75)	27.97(18.23,41.75)	0.857
Changes in the diameter, P50(P25,P75),%	33.81(19.90,49.37)	39.42(18.63,57.22)	0.267
Therapeutic evaluation			0.742
Partial Remission	63(58.3%)	61(63.5%)	
Stable Disease	42(38.9%)	32(33.3%)	
Progression Disease	3(2.8%)	3(3.1%)	
Adverse events			0.78
III	10(9.3%)	6(6.3%)	
IV	1(0.9%)	1(1%)	

Based on the clinical stage, there was no statistically significant difference in the distribution of tumor stages between the two groups (*p* = 0.507). The majority of patients in both groups were classified as stage IIIA, with 40 (37%) in the < = 33 days group and 29 (30.2%) in the > 33 days group. There were no statistically significant differences in the number of treatment cycles (*p* = 0.58) or the choice of immunotherapy drugs (*p* = 0.139) between the two groups. Following neoadjuvant immunochemotherapy, there was no statistically significant difference in the therapeutic evaluation between the two groups (*p* = 0.742), with 63 (58.3%) individuals achieving PR in the < = 33 days group and 61 (63.5%) in the > 33 days group. The ORR was 58.3% in the < = 33 days group and 63.5% in the > 33 days group, with no statistically significant difference (*p* = 0.447). Regarding adverse events between the two groups, there was no statistically significant difference in Grade III adverse events, with 10 (9.3%) individuals in the < = 33 days group and 6 (6.3%) in the > 33 days group (*p* = 0.78). The main reasons for these adverse events included blood cell reduction (11 individuals), liver impairment (3 individuals), skin and mucous membrane reactions (1 individual), and gastrointestinal reactions (1 individual). There was one individual in each group with Grade IV adverse events, accounting for 0.9% and 1% respectively, and both cases were due to granulocyte reduction (2 individuals). Comprehensive data on neoadjuvant immunochemotherapy is detailed in Table 2.

**Table 2:** Surgical-related information

Variables	Group		*p*
	<=33d	> 33d	
**Surgical margin**			0.28
R0	103(95.4%)	88(91.7%)	
R1	5(4.6%)	8(8.3%)	
**Tumor location**			0.767
Inferior lobe of left lung	31(28.7%)	24(25%)	
Superior lobe of left lung	16(14.8%)	19(19.8%)	
Hilum of left lung	2(1.9%)	0(0%)	
Inferior lobe of right lung	31(28.7%)	29(30.2%)	
Middle lobe of right lung	5(4.6%)	7(7.3%)	
Superior lobe of right lung	22(20.4%)	16(16.7%)	
Hilum of right lung	1(0.9%)	1(1%)	
Operation			0.748
Wedge resection	1(0.9%)	1(1%)	
Pulmonary segmental resection	0(0%)	1(1%)	
Pulmonary lobectomy	48(44.4%)	48(50%)	
Pulmonary sleeve resection	36(33.3%)	31(32.3%)	
Total pneumonectomy	5(4.6%)	5(5.2%)	
Open operation	18(16.7%)	10(10.4%)	
Whether to convert to open surgery during the operation			0.007
No	81(75%)	86(89.6%)	
Yes	27(25%)	10(10.4%)	
Duration of surgery, P50(P25,P75),min	156(122.5,210)	155(126.5,209)	0.88
Amount of bleeding, P50(P25,P75),ml	50(22.5,100)	50(20,100)	0.349
Number of lymph node dissection, P50(P25,P75)	20(14,29)	19(12,27)	0.15
Hospital stays, P50(P25,P75),d	7(5,10)	7(5,9)	0.509
Postoperative treatment			0.427
90-day mortality after surgery, (%)	0	1(1.04)	0.288
**yp-stage**			0.377
0	21(19.4%)	19(19.8%)	
IA	45(41.7%)	34(35.4%)	
IB	9(8.3%)	4(4.2%)	
IIA	3(2.8%)	2(2.1%)	
IIB	16(14.8%)	22(22.9%)	
IIIA	14(13%)	12(12.5%)	
IIIB	0(0)	3(3.1%)	
MPR			0.569
No	65(60.2%)	54(56.3%)	
Yes	43(39.8%)	42(43.8%)	
PCR			0.442
No	83(76.9%)	78(81.3%)	
Yes	25(23.1%)	18(18.8%)	

The surgical approaches did not show any statistically significant difference between the two groups (*p* = 0.748). The most common surgical procedure in both groups was pulmonary lobectomy, with 48 (44.4%) in the < = 33 days group and 48 (50%) in the > 33 days group.In the < = 33 days group, 103 (95.4%) individuals achieved R0 resection, while in the > 33 days group, there were 88 (91.7%) individuals. This difference was not statistically significant (*p* = 0.28).It was observed that in the < = 33 days group, there were more cases where minimally invasive surgeries were converted to open surgeries compared to the > 33 days group, and this difference was statistically significant [27 (25%) individuals vs. 10 (10.4%) individuals, *p* = 0.007]. The number of lymph node dissection did not show any statistically significant difference between the two groups (*p* = 0.15). The median length of hospital stay was 7 days for both groups, and there was no statistically significant difference (*p* = 0.509). For further details regarding surgical-related information, please refer to Table 3.

**Table 3:** Cox regression analysis for DFS

Variables	Univariate analysis		Multivariate analysis	
	HR (95% CI)	*P* value	HR (95% CI)	*P* value
Treatment of interval		0.002		0.004
<=33d	0.368(0.194, 0.7)		0.347(0.168, 0.717)	
> 33d	Reference		Reference	
BMI	0.874(0.781, 0.977)	0.018	0.873(0.774, 0.985)	0.027
Maximum diameter of tumor after treatment	1.017(1.001, 1.033)	0.04	1.023(1.005, 1.042)	0.013
Surgical margin		0.042		0.987
R0	0.375(0.145, 0.965)		0.990(0.310, 3.165)	
R1	Reference		Reference	
Tumor location		0.033		0.067
Inferior lobe of left lung	0.104(0.023, 0.477)	0.004	0.061(0.009, 0.412)	0.004
Superior lobe of left lung	0.088(0.018, 0.44)	0.003	0.007(0.011, 0.480)	0.007
Hilum of left lung	0(0, 7.94E + 254)	0.967	0.000(0, -)	0.973
Inferior lobe of right lung	0.078(0.016, 0.37)	0.001	0.052(0.008, 0.325)	0.002
Middle lobe of right lung	0.249(0.047, 1.312)	0.101	0.183(0.029, 1.134)	0.068
Superior lobe of right lung	0.113(0.023, 0.543)	0.007	0.079(0.011, 0.545)	0.010
Hilum of right lung	Reference		Reference	
Whether to convert to open surgery during the operation		0.016		0
No	0.424(0.21, 0.855)		0.079(0.011, 0.545)	
Yes	Reference		Reference	
yp-stage		0		-
0	0.062(0.009, 0.44)	0.005		-
IA	0.095(0.02, 0.447)	0.003		-
IB	0.05(0.004, 0.565)	0.015		-
IIA	0(0, .)	0.974		-
IIB	0.45(0.102, 1.99)	0.293		-
IIIA	0.831(0.185, 3.719)	0.808		-
IIIB	Reference			
MPR		0.011		0.516
No	2.714(1.256, 5.867)		1.370(0.530, 3.538)	
Yes	Reference		Reference	
PCR		0.018		0.171
No	5.565(1.343, 23.057)		3.419(0.589, 19.864)	
Yes	Reference		Reference	

**Fig. 2:**
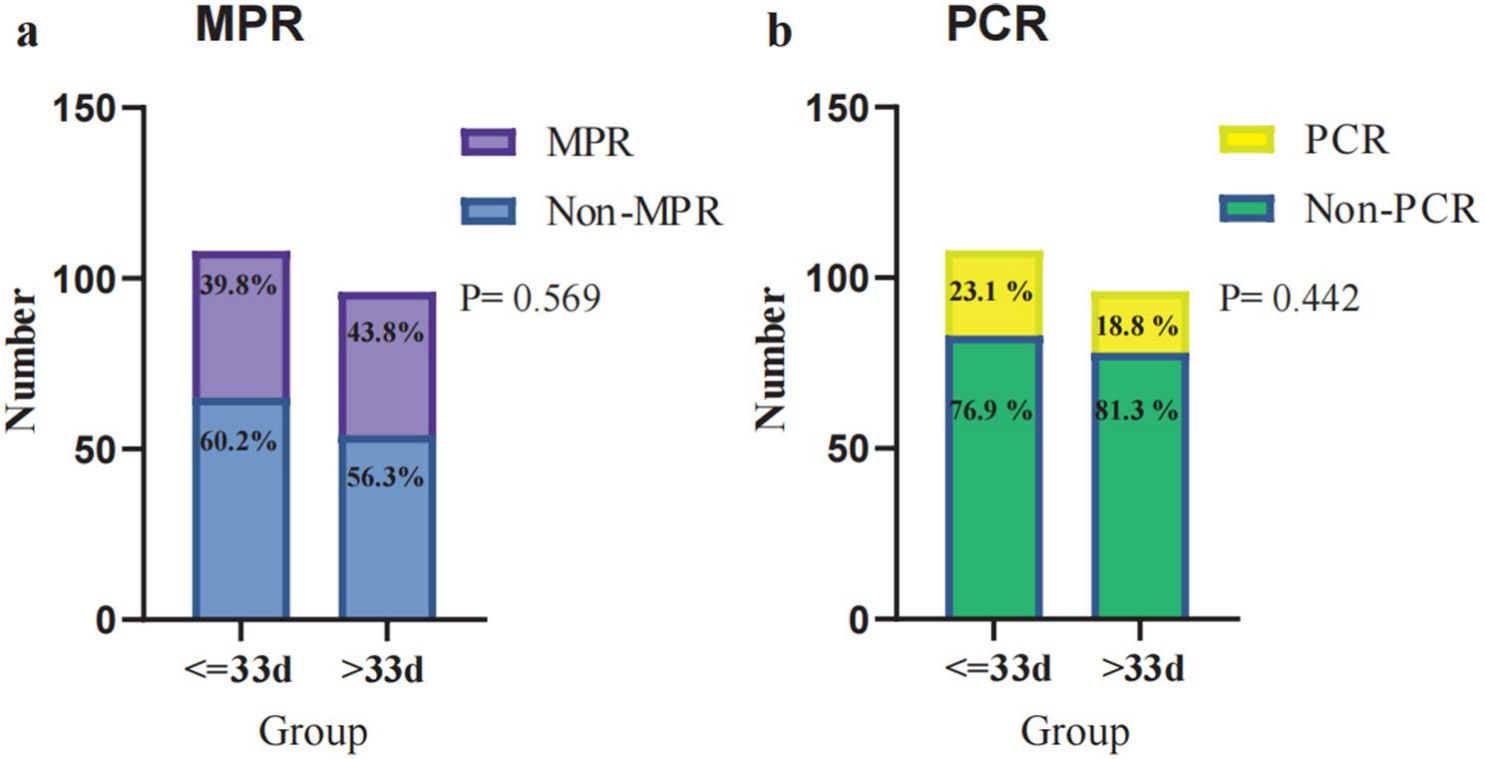
(**a**) Tumor MPR was achieved with 43 (39.8%) and 42 (43.8%) in the two groups, respectively, *p* = 0.569. (**b**) PCR was achieved with 25 (23.1%) and 18 (18.8%) in both groups, *p* = 0.442. Major Pathological Response, MPR. Pathological Complete Remission, PCR

Based on yp-stage, there was no statistically significant difference in tumor staging between the two groups (*p* = 0.337). The majority of patients in both groups were classified as yp-stage IA, with 45 (41.7%) individuals in the < = 33 days group and 34 (35.4%) individuals in the > 33 days group. In terms of achieving Tumor MPR, there were 43 (39.8%) individuals in the < = 33 days group and 42 (43.8%) individuals in the > 33 days group, with no statistically significant difference (*p* = 0.569). Similarly, in achieving pCR, there were 25(23.1%) individuals in the < = 33 days group and 18(18.8%) individuals in the > 33 days group, again with no statistically significant difference (*p* = 0.442)(Fig. 2). Pathological details can be found in Table 2. The results of the logistic regression univariate analysis analysis indicated that the treatment interval does not impact the pathological response of tumors and lymph nodes (Supplementary Table 1). The 90-day postoperative mortality rates were 0% and 1.04%, respectively, with no statistical difference. A total of 31 people experienced recurrence or metastasis. In the < = 33d group, 11 people (10.2%) were affected, of which 5 people (4.6%) had local recurrence and 6 people (5.6%) had distant metastasis. In the > 33d group, 20 people (20.8%) were affected, of which 12 people (12.5%) had local recurrence and 8 people (8.3%) had distant metastasis.

**Fig. 3:**
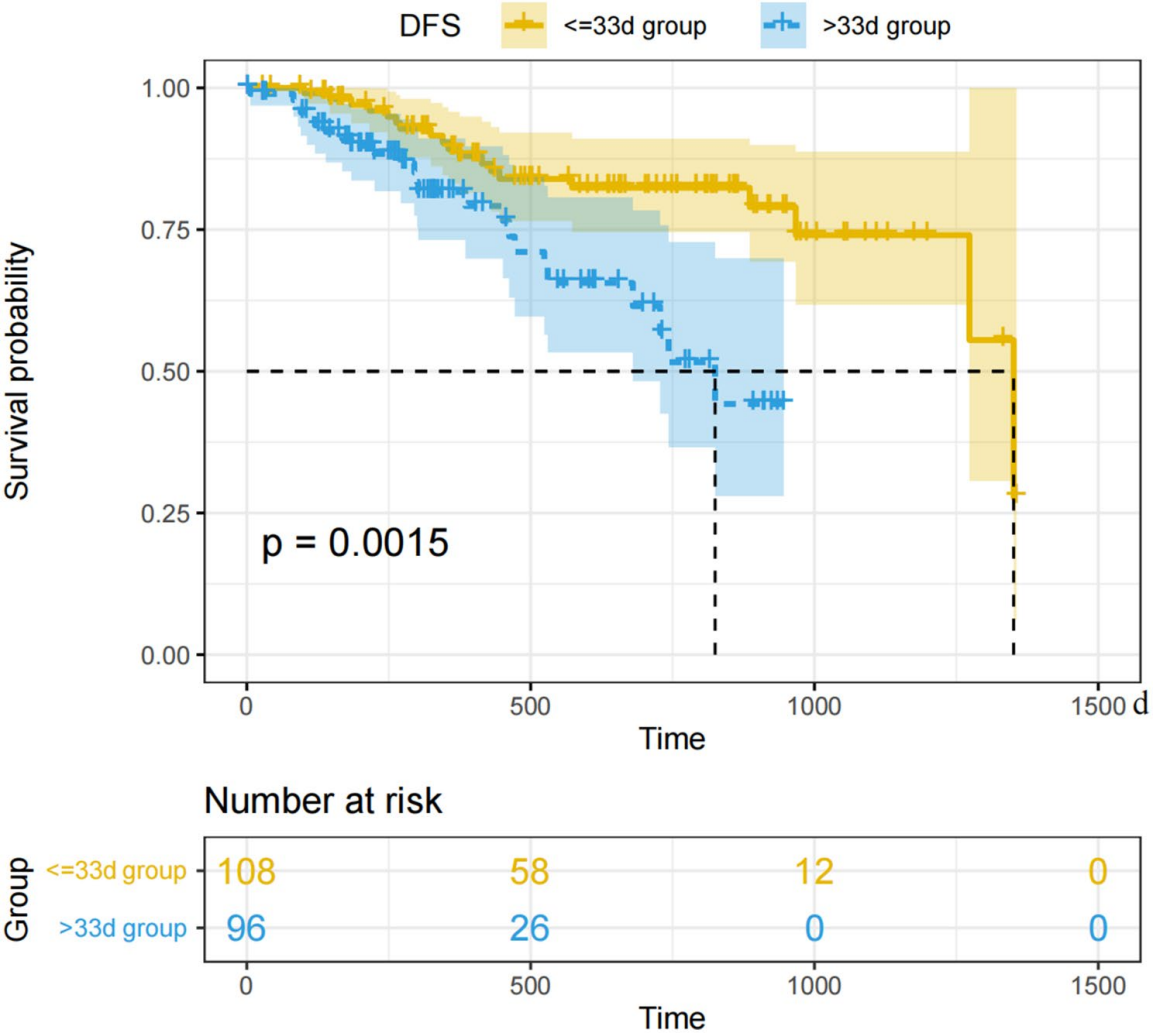
Survival curves for Disease-Free Survival

**Fig. 4:**
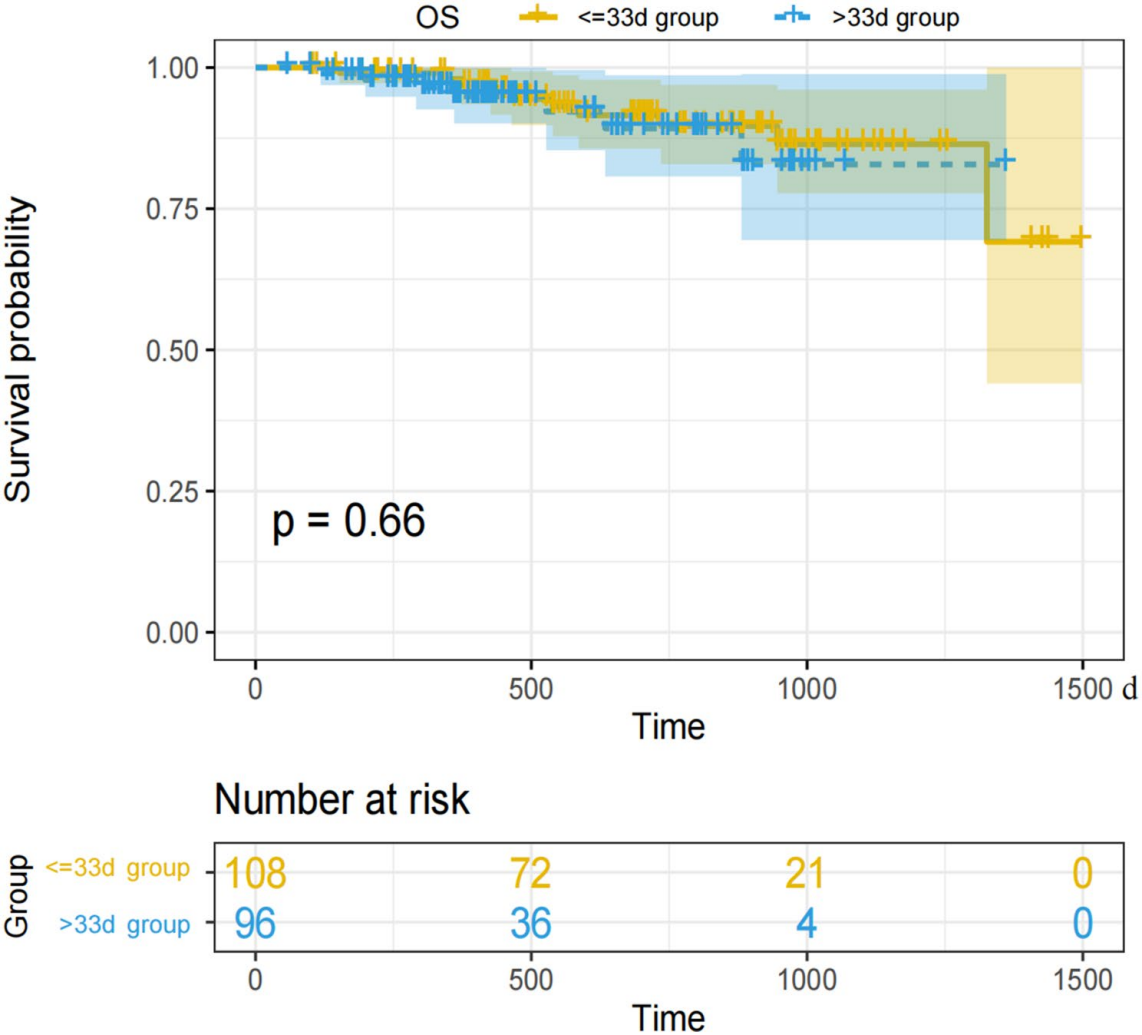
Survival curve for overall survival

Based on the Kaplan-Meier methods, the < = 33 days group exhibited a better DFS curve compared to the > 33 days group (*p* = 0.0015) (Fig. 3). The median DFS for the two groups was 952 days and 590 days, respectively. However, there was no statistically significant difference in the OS curves between the two groups (*p* = 0.66), and the median OS was not reached (Fig. 4).

In the Cox regression univariate analysis analysis for DFS, treatment interval < = 33d (HR = 0.368(0.194, 0.7), *p* = 0.002), BMI(HR = 0.874(0.781, 0.977), *p* = 0.018), Maximum diameter of tumor after treatment (HR = 1.017(1.001, 1.033), *p* = 0.04) R0resection (HR = 0.375(0.145, 0.965), *p* = 0.042), Tumor location(*p* = 0.033, Without converting to open surgery during the operation (HR = 0.424(0.21, 0.855), *p* = 0.016),yp-stage(*p* < 0.001, non-MPR (HR = 2.714(1.256, 5.867), *p* = 0.011), non-pCR(HR = 5.565(1.343, 23.057), *p* = 0.018) have statistically significant difference. In the multivariate analysis, treatment interval < = 33d (HR = 0.347(0.168, 0.717), *p* = 0.004), BMI (HR = 0.873(0.774, 0.985), *p* = 0.027), Maximum diameter of tumor after treatment (HR = 1.023(1.005, 1.042), *p* = 0.027), Without converting to open surgery during the operation (HR = 0.079(0.011, 0.545), *p* = 0) have statistically significant difference(Table 3).

## Discussion

Our research found that the treatment interval affects DFS in LUSC, with patients who had shorter treatment intervals experiencing better DFS outcomes. It was observed that patients with shorter treatment intervals exhibited a slightly better OS curve in some instances, despite lacking statistical significance. Omarini et al. [18] found that shorter treatment intervals after neoadjuvant chemotherapy correlated with better OS and Recurrence-Free Survival in the patients with breast cancer .This is similar to our results. We will continue to monitor the subsequent survival of the patients in this study.

Additionally, this study observed that the treatment interval does not impact MPR or pCR. There were no statistically significant differences between the two groups in this regard.This is consistent with previous research findings [19].With similar baseline characteristics, it was observed that the treatment interval did not affect the duration of surgery, the amount of bleeding, or the length of hospital stay. The findings suggest that undergoing surgery with a shorter treatment interval is safe.

There was no statistical difference in the adverse reactions caused by neoadjuvant treatment between the two groups of patients, indicating that the patients’ physical condition may not affect the differences of treatment interval and long-term outcomes. Patients were evaluated for surgical indications by the primary physician through a Multidisciplinary Team assessment 3–4 weeks after the completion of the last neoadjuvant treatment and then took about a week to complete the hospital admission and surgical procedures. This may result in variations in the treatment intervals for the patients. At the same time, due to patients’ hesitancy about surgery, the time spent on re-examinations, scheduling surgery, and other personal reasons, some patients had a longer treatment interval.

In this study, operation had a higher proportion of conversion to open surgery with a shorter treatment interval. Patients after neoadjuvant therapy might experience changes such as tissue edema, destruction of tissue gap structures, increased fragility of capillaries, and tissue adhesion caused by tumor shrinkage [20, 21].We believe that appropriately extending the treatment interval might allow tissue edema to subside and interstitial spaces to reform, enabling surgeons to maintain the original ooperation. Additionally, due to the sample size differences in the variables of whether to convert to open surgery during the operation, the stage, and pCR rate, we believe this might have influenced the results in the Cox regression. ypStage as well as MPR/PCR are confounding factors. Therefore, the ypStage variable was not included in the multivariate analysis. In the univariate analysis, Tumor MPR, pCR have a positive impact on DFS. This provides evidence for whether MPR and pCR can serve as surrogate endpoints in survival analysis. In this study, the proportion of males was significantly higher than that of females. Upon reviewing medical records, we found that this might be because many female patients had adenocarcinoma with gene mutations and ultimately underwent targeted therapy. Many patients with stage IB/IIA, due to comorbidities, were temporarily unable to undergo surgical treatment or chose neoadjuvant treatment due to hesitancy about surgery.

Studies investigating the treatment interval for other types of tumors have yielded varying results. Du et al. [22] suggests that prolonging the treatment interval (> 8 weeks) in neoadjuvant chemoradiotherapy can improve the pCR rate in rectal cancer. Sanford et al. [23]suggests that delaying breast cancer surgery by more than 8 weeks after neoadjuvant chemotherapy can have a negative impact on OS. These studies used different approaches of neoadjuvant therapy, which may have influenced the results. However, the effect of treatment interval in neoadjuvant immunochemotherapy should be paid more attention.

Liu et al. [4] discovered that there was an increased proportion of IFN producing lung tumor-specific T cells in neoadjuvant immunotherapy with shorter treatment intervals. Previous studies have demonstrated that the efficacy of neoadjuvant immunotherapy relies on CD8 + T cells and IFN [24]. They propose that the timely removal of the primary tumor at the height of tumor-specific T cell expansion. During neoadjuvant immunotherapy, the appropriate timing for primary tumor resection might play a crucial role in the expansion and functionality of tumor-specific T cells (especially gp70 tetramer-specific CD8 T cells). The primary tumor serves as an essential source of tumor antigens, housing a significant number of tumor-specific gp70-T cells. If these cells remain in the primary tumor for an extended period, they might become functionally impaired or exhausted, losing their ability to target tumor cells. Exhausted T cells may exhibit increased inhibitory receptors (such as PD-1) and might not effectively respond to tumor antigens. By resecting the primary tumor at the right moment, these cells can be prevented from being trapped within the tumor. This allows them to migrate to other parts of the body, such as metastatic sites, enhancing the efficacy of neoadjuvant immunotherapy and potentially improving long-term survival rates. This provides some theoretical support for the impact of treatment intervals on survival. However, the mechanisms underlying the relationship between treatment intervals and survival outcomes in neoadjuvant immunochemotherapy are not yet fully understood. Further research is needed to elucidate this issue.

In clinical practice, treatment intervals might not have received the attention they deserve, and there might not be standardized guidelines in place. However, patients could experience anxiety due to extended periods without undergoing surgery. Additionally, treatment intervals could potentially impact survival outcomes. We have identified and reported this clinical phenomenon, although the specific mechanisms are not yet clear. In the future, more research is needed in the future to study the optimal treatment timing, and it’s crucial to enhance patient education and communication regarding treatment intervals and their potential implications. In clinical practice, it’s necessary to reduce delays in treatment intervals caused by hospital admission procedures and individual patient reasons.

## Conclusion

This study found that shorter treatment intervals were associated with better DFS. However, treatment intervals did not affect OS, pathological response, or surgical safety. Patients should avoid having a prolonged treatment interval between neoadjuvant immunochemotherapy and surgery.

## Limitations

This study is a single-center retrospective study with a limited sample size. Future research should encompass multi-center studies to mitigate selection biases.

### Electronic supplementary material

Below is the link to the electronic supplementary material.

Supplementary Material 1

Supplementary Material 2

## Data Availability

The datasets generated and/or analysed during the current study are not publicly available due [REASON WHY DATA ARE NOT PUBLIC] but are available from the corresponding author on reasonable request.

## Abbreviations

DFS: Disease-free survival
OS: Overall survival
ORR: Objective response rate
MPR: Major pathological response
pCR: Pathological complete remission
LUSC: lung squamous cell carcinoma
PD-1: programmed cell death protein 1
CR: Complete remission
PR: Partial remission
PD: Progression disease
SD: Stable disease

## References

[1] Travis WD (2020). Lung Cancer Pathology: current concepts. Clin Chest Med.

[2] Lahiri A, Maji A, Potdar PD, Singh N, Parikh P, Bisht B (2023). Lung cancer immunotherapy: progress, pitfalls, and promises. Mol Cancer.

[3] Gao S, Li N, Gao S, Xue Q, Ying J, Wang S (2020). Neoadjuvant PD-1 inhibitor (Sintilimab) in NSCLC. J Thorac Oncology: Official Publication Int Association Study Lung Cancer.

[4] Liu J, O’Donnell JS, Yan J, Madore J, Allen S, Smyth MJ (2019). Timing of neoadjuvant immunotherapy in relation to surgery is crucial for outcome. Oncoimmunology.

[5] Provencio M, Nadal E, Insa A, Garcia-Campelo MR, Casal-Rubio J, Domine M (2020). Neoadjuvant chemotherapy and nivolumab in resectable non-small-cell lung cancer (NADIM): an open-label, multicentre, single-arm, phase 2 trial. Lancet Oncol.

[6] Sepesi B, Zhou N, William WN, Lin HY, Leung CH, Weissferdt A (2022). Surgical outcomes after neoadjuvant nivolumab or nivolumab with ipilimumab in patients with non-small cell lung cancer. J Thorac Cardiovasc Surg.

[7] Zhu X, Sun L, Song N, He W, Xie B, Hu J (2022). Safety and effectiveness of neoadjuvant PD-1 inhibitor (toripalimab) plus chemotherapy in stage II-III NSCLC (LungMate 002): an open-label, single-arm, phase 2 trial. BMC Med.

[8] Cascone T, Leung CH, Weissferdt A, Pataer A, Carter BW, Godoy MCB (2023). Neoadjuvant chemotherapy plus nivolumab with or without ipilimumab in operable non-small cell lung cancer: the phase 2 platform NEOSTAR trial. Nat Med.

[9] Shu CA, Gainor JF, Awad MM, Chiuzan C, Grigg CM, Pabani A (2020). Neoadjuvant atezolizumab and chemotherapy in patients with resectable non-small-cell lung cancer: an open-label, multicentre, single-arm, phase 2 trial. Lancet Oncol.

[10] Forde PM, Chaft JE, Smith KN, Anagnostou V, Cottrell TR, Hellmann MD (2018). Neoadjuvant PD-1 blockade in Resectable Lung Cancer. N Engl J Med.

[11] Goldstraw P, Chansky K, Crowley J, Rami-Porta R, Asamura H, Eberhardt WE (2016). The IASLC Lung Cancer Staging Project: proposals for revision of the TNM Stage groupings in the Forthcoming (Eighth) Edition of the TNM classification for Lung Cancer. J Thorac Oncology: Official Publication Int Association Study Lung Cancer.

[12] Charlson ME, Pompei P, Ales KL, MacKenzie CR (1987). A new method of classifying prognostic comorbidity in longitudinal studies: development and validation. J Chronic Dis.

[13] Charlson M, Szatrowski TP, Peterson J, Gold J (1994). Validation of a combined comorbidity index. J Clin Epidemiol.

[14] NCI. *NCI (2017). Common Terminology criteria for adverse Events (CTCAE) version 5.0. NCI*.

[15] Weissferdt A, Pataer A, Vaporciyan AA, Correa AM, Sepesi B, Moran CA (2020). Agreement on major pathological response in NSCLC patients receiving Neoadjuvant Chemotherapy. Clin Lung Cancer.

[16] Wang H, Mao X (2020). Evaluation of the efficacy of neoadjuvant chemotherapy for breast Cancer. Drug Des Devel Ther.

[17] Deng H, Zhao Y, Cai X, Chen H, Cheng B, Zhong R (2022). PD-L1 expression and tumor mutation burden as pathological response biomarkers of neoadjuvant immunotherapy for early-stage non-small cell lung cancer: a systematic review and meta-analysis. Crit Rev Oncol/Hematol.

[18] Omarini C, Guaitoli G, Noventa S, Andreotti A, Gambini A, Palma E (2017). Impact of time to surgery after neoadjuvant chemotherapy in operable breast cancer patients. Eur J Surg Oncol.

[19] Chen J, Deng H, He J, Wang Z, Li S (2022). Impact of the interval between neoadjuvant immunochemotherapy and surgery on surgical-pathological outcomes in non-small cell lung cancer. Front Oncol.

[20] Liang H, Yang C, Gonzalez-Rivas D, Zhong Y, He P, Deng H (2021). Sleeve lobectomy after neoadjuvant chemoimmunotherapy/chemotherapy for local advanced non-small cell lung cancer. Transl Lung Cancer Res.

[21] Ma S, Yan T, Liu D, Wang K, Wang J, Song J (2018). Neoadjuvant chemotherapy followed by minimally invasive esophagectomy is safe and feasible for treatment of esophageal squamous cell carcinoma. Thorac Cancer.

[22] Du D, Su Z, Wang D, Liu W, Wei Z (2018). Optimal interval to surgery after neoadjuvant chemoradiotherapy in rectal Cancer: a systematic review and Meta-analysis. Clin Colorectal Cancer.

[23] Sanford RA, Lei X, Barcenas CH, Mittendorf EA, Caudle AS, Valero V (2016). Impact of Time from Completion of Neoadjuvant Chemotherapy to surgery on survival outcomes in breast Cancer patients. Ann Surg Oncol.

[24] Liu J, Blake SJ, Yong MC, Harjunpää H, Ngiow SF, Takeda K (2016). Improved efficacy of Neoadjuvant compared to Adjuvant Immunotherapy to Eradicate Metastatic Disease. Cancer Discov.

