# Supplementary material for: Impact of treatment interval between neoadjuvant immunochemotherapy and surgery in lung squamous cell carcinoma
Source: BMC Cancer. 2024 May 13;24:585. doi: 10.1186/s12885-024-12333-3 (PMC11089690; doi:10.1186/s12885-024-12333-3)
Supplement: Supplementary file 1 — Supplementary Material 1 [file 12885_2024_12333_MOESM1_ESM.doc]

| **Tumor MPR** |  |  |
| --- | --- | --- |
| Variables | Univariate analysis | |
|  | OR (95% CI) | P value |
| Treatment of interval | | 0.569 |
| <=33d | Reference |  |
| >33d | 0.851（0.487，1.486） |  |
|  |  |  |
|  |  |  |
| **PCR** |  |  |
| Variables | Univariate analysis | |
|  | OR (95% CI) | P value |
| Treatment of interval | | 0.443 |
| <=33d | Reference |  |
| >33d | 0.766（0.388，1.513） |  |

Supplementary table 2: Treatment interaval ‘s effect on pathological response.
