# Supplementary material for: Impact of treatment interval between neoadjuvant immunochemotherapy and surgery in lung squamous cell carcinoma
Source: BMC Cancer. 2024 May 13;24:585. doi: 10.1186/s12885-024-12333-3 (PMC11089690; doi:10.1186/s12885-024-12333-3)
Supplement: Supplementary file 2 — Supplementary Material 2 [file 12885_2024_12333_MOESM2_ESM.doc]

| Variables | Group | | p |
| --- | --- | --- | --- |
|  | <=33d | >33d |  |
| Treatment interval，P50(P25,P75),d | 29(27,31) | 38(35,47) | 0 |
| Sex |  |  | 0.668 |
| Male | 106（98.1%） | 93（96.9%） |  |
| Female | 2（1.9%） | 3（3.1%） |  |
| Age，P50(P25,P75),y | 65(60,71) | 66(60,69) | 0.575 |
| ECOG |  |  | 0.578 |
| 0 | 77（71.3%） | 65（67.7%） |  |
| 1 | 31（28.7%） | 31（32.3%） |  |
| BMI，mean（SD）,kg/m^2 | 22.52(2.736) | 22.57(3.049) | 0.902 |
| Smoking history |  |  | 0.171 |
| No | 34（31.5%） | 22（22.9%） |  |
| Yes | 74（68.5%） | 74（77.1%） |  |
| Hypertension |  |  | 0.395 |
| No | 79（73.1%） | 65（67.7%） |  |
| Yes | 29（26.9%） | 31（32.3%） |  |
|  |  |  |  |
| Diabetes mellitus |  |  | 0.587 |
| No | 94（87%） | 81（84.4%） |  |
| Yes | 14（13%） | 15（15.6%） |  |
| Cardiac disease |  |  | 0.099 |
| No | 100（92.6%） | 82（85.4%） |  |
| Yes | 8（7.4%） | 14（14.6%） |  |
| Cancer history |  |  | 0.833 |
| No | 102（94.4%） | 90（93.8%） |  |
| Yes | 6（5.6%） | 6（6.3%） |  |
| Chronic obstructive pulmonary disease | |  | 0.748 |
| No | 95（88%） | 83（86.5%） |  |
| Yes | 13（12%） | 13（13.5%） |  |
| aCCIs |  |  | 0.627 |
| 0-1 | 19（17.6%） | 17（17.7%） |  |
| 2-3 | 74（68.5%） | 61（63.5%） |  |
| ≥4 | 15（13.9%） | 18（18.8%） |  |
| Maximum diameter of the initial tumor，P50(P25,P75),mm | 47(34,60) | 47(34,67) | 0.359 |

Supplementary table 1: Baseline information about patients.
